# Appendicitis—When Not to Operate*Read before the Kansas City Academy of Medicine, November 12, 1898.

**Published:** 1899-01

**Authors:** Milo B. Ward


### APPENDICITIS—WHEN NOT TO OPERATE.*

BY MILO B. WARD, M.D.

Successful management of appendicitis requires mature judgment, extensive experience in operations on intra-abdominal organs, and an acquaintance with the etiology, symptoms, and pathology of this disease, which can be possessed by those only who have had unusual opportunities of observing every feature of the medical and surgical aspect of this affection, and the history of the various methods of treatment.

There is at present a tendency to establish the claim that surgeons who are familiar with the good results to be obtained from surgery in appendicitis are *always* and *invariably opposed* to palliative measures, and do not see any good to be derived from treatment by medicines, nursing, rest, etc. In order to make himself thoroughly understood on this subject the writer has considered it worth while to present his personal views regarding the non-operative measures in some cases and under some circumstances. It would be wasting your valuable time for the writer to dilate on his *unwavering* faith in surgery as the rational treatment of suppurating appendicitis, when suitable conditions can be demonstrated with reasonable certainty.

We are occasionally called upon to see a patient whose condition at the time of first observation is such that operative procedure is unwise and possibly would increase the mortality. This class of cases requires extreme caution, and experience has convinced the writer of the wisdom of insisting on the expectant plan of treatment in all cases where the manipulation of the abdominal viscera would add to the shock which may be already quite severe.

It is matter of much surprise that there is such a marked difference of opinion regarding the operative and non-operative treatment of diseases of the appendix. It should not be so difficult to comprehend the apparent contradiction. Equally good, conscientious and skillful men may be found on either side of the question. The trouble is that we are prone to be entirely and unalterably *opposed* to surgery or in *favor* of it. We must learn once for all that there is virtue in both the expectant and the purely surgical plans of treatment. When we have mastered this truth, we will have settled much of the controversy, bridged over the chasm, and united the medicine-man and the surgical-man by a bond of mutual assistance that will result in great good to the profession and suffering humanity.

---

*Read before the Kansas City Academy of Medicine, November 12, 1898.

It is not to be expected that the general practitioner who has had occasion to see the mortality following surgery for this affliction is going to call in consultation a surgeon who *invariably* recommends surgery, regardless of the circumstances. The percentage of recoveries from the attacks of diagnosed appendicitis without surgery is so large that the members of the profession can not ignore the fact, and whenever a surgeon says that surgery alone promises relief in *every* case, he is giving an opinion so directly contrary to the established fact, that the cause of surgery is harmed, and the attending physicians are so frightened that they hesitate to summon surgeons when they really find the need of their extensive experience in the treatment of this obscure disease.

If the foregoing statements are tenable, then we may make the rule that, *all cases of appendicitis should be treated by the phycician and surgeon together.* This not only divides the responsibility but it gives the patient the benefit of the experience of two or more physicians; the well-equipped general practitioner and the well-rounded surgeon whose experience should not in the least detract from the good sense acquired in the practice of his profession in former years as a general practitioner.

It is timely to add further that the surgeon who neglects the study and investigation of the medical aspect of the treatment of human ailments, would soon discover himself scrambling to get into the procession which has already gone by him.

Some one has facetiously remarked that the true surgeon avoids the knife, but he knows how to use it when the occasion requires. Is it not apparent that the tendency among those of us who do nothing but surgery is to see surgical indications and shut our eyes to all other means of relief, when, as a matter of fact, we may be mistaken in our judgment and obliged to face the chagrin that follows?

Is it not better to make a reservation in doubtful cases, and admit that some patients recover temporarily and for all we know sometimes permanently, without surgery? This is the view of the problem which the writer has had forced upon him by an experience which, although somewhat limited, as compared with others, yet is of sufficient scope to furnish him many examples to corroborate the statement made by others more experienced, namely: that proper medication, extreme quietude, external applications of cold or heat, and abstinence from food of every kind for a considerable period of time will accomplish wonders, when surgery can not be safely done. Do not misunderstand the writer's assertion. This method is not to take place of surgery if surgery is indicated, and will give reasonable promise of success, but rather to be resorted to when indications and environments contraindicate surgical measures for the time being, for our duty is to rescue the patient from imminent danger. After the graver symptoms have passed, surgery is nearly always indicated to give assurance of permanent recovery.

The obscure nature of this disease has been the most potent factor in the controversy between the surgeon and the physician. If every case of threatened appendicitis could readily be diagnosed, then the remedy would be plain, provided, of course, that the remedy suggested meets the approval of those interested. How frequently it happens when the attending physician informs the friends that, in his opinion, the patient is suffering from appendicitis and requests the privilege of calling an abnominal surgeon, with a view of operation, he is denied this request for fear that surgery will be recommended any way, regardless of conditions. Would it not be infinitely better for all concerned, were it possible for the physician to inform the patient and friends that they are mistaken regarding their opinion of the surgeon, that, as a matter of fact, he, the surgeon, is just as reluctant to recommend surgery as possible, and there need be no fear of surgery unless this is the wisest and safest means in the case under consideration?

In a discussion of the subject of appendicitis at a recent meeting of the American Medical Association held at Denver, a very prominent practitioner said, "In cases of appendicitis I want a skilled and competent surgeon, not for operating, but for his judgment." *Is this not the key the whole question?* This same physician says if he is satisfied that appendicitis exists he is willing that the appendix should be removed.

Dr. H. D. Niles, of Salt Lake, in a very able paper read at Denver, asks this question, "Are experienced operators ever justified in recommending that an infected appendix be permitted to remain within the abdomen longer than is needed to make the necessary preparations for its removal?" The writer's reply is *No*, but we must qualify this answer by admitting that *Time* is an essential factor in the preparation.

If the patient's condition does not justify an immediate operation, it is good surgery not to operate until the symptoms which are present and make the operation too hazardous have subsided, and this will be in keeping with the doctrine of *operating as soon as it can be done at all safely.*

The condition of the patient and the environment (and what is implied by environment includes the prospective after care of the patient) are questions to be settled before an operation can be insisted upon as the *only remedy.*

If the patient is seen by the surgeon early in the history of the attack, he will not be hampered by conditions which are nearly certain to be present later in the case. While it is true that the surroundings may be the same, the condition of the patient is such that it will not matter so much what the surroundings are. If called to see the patient when there is great distention of the intestine, and the patient is already in shock, it is infinitely wiser to postpone surgery, for in all probability, surgery will avail nothing, and *indirectly do great harm.*

It can not be truthfully claimed that the writer is a timid operator or cowardly in his dealings with grave conditions, but his experience has taught that "discretion is the better part of valor," and surgeons should not always venture where angels dare not go.

If the surgeon is called into the case in the early history he should be held more than the physician responsible for the grave after condition of the patient, unless it be true that his recommendations were not carried out, for it is to be supposed that he has given the opinion that surgery is the logical treatment for suppurating appendicitis, when once the diagnosis is assured.

It is also to be supposed that the surgeon has informed the patient and friends that delay, when the indications are proper for operation, may lead to conditions when neither surgeon nor human skill under any circumstances can save life.

An attempt to make a "pen picture" of the conditions which contraindicate surgical procedure for appendicitis, would be superfluous.

*Surgical acumen will serve as the guiding star.—Kansas City Medical Lancet.*

